# Estimation of Sow Backfat Thickness Based on Machine Vision

**DOI:** 10.3390/ani14233520

**Published:** 2024-12-05

**Authors:** Yue Jian, Shihua Pu, Jiaming Zhu, Jianlong Zhang, Wenwen Xing

**Affiliations:** 1 Chongqing Academy of Animal Sciences, Chongqing 402460, China; jiany16@163.com (Y.J.);; 2 College of Biosystems Engineering and Food Science, Zhejiang University, 866 Yuhangtang Road, Hangzhou 310058, China; 3 Key Laboratory of Intelligent Equipment and Robotics for Agriculture of Zhejiang Province, Hangzhou 310058, China; 4 College of Agricultural Engineering, Shanxi Agricultural University, Taigu 030801, China

**Keywords:** backfat thickness, sows, LabVIEW, machine vision, buttock morphological parameters

## Abstract

Controlling the backfat thickness of sows within an appropriate range during different production stages helps to increase the number of pigs weaned per sow per year and ultimately enhances the economic benefit to the pig farm. To obtain the backfat thickness of sows automatically, a backfat thickness estimation method based on machine vision is proposed. First, the backfat thickness values and 3D images of the buttocks of 154 Landrace–Yorkshire crossbred sows were obtained using a veterinary ultrasound backfat meter and Azure Kinect DK camera. After preprocessing the 3D images utilizing Python 3.9.16 software, 10 external morphological parameters reflecting the area, width, height, and contour radius of the sow’s buttocks were extracted. The relationships between backfat thickness and external morphological parameters were analyzed in a randomly selected group of 100 sows. A significant positive correlation was observed between backfat thickness and buttock morphological parameters, with the Pearson coefficient for the fitted ellipse area achieving values up to 0.90. A backfat thickness estimation model was developed based on selected buttock feature parameters. The model’s generalization performance was evaluated using 54 additional sows that were not involved in the model development. The coefficient of determination (R^2^) between the estimated and actual backfat thicknesses was 0.8923, with a mean absolute error (MAE) of 1.23 mm and a mean absolute percentage error (MAPE) of 5.73%. These metrics indicate that the model can meet production requirements, and the proposed technique offers improved estimation accuracy compared to existing methods. Ultimately, a backfat thickness automatic estimation system was developed using LabVIEW 2023 Q1 (64-bit) software. This research helps to address the cumbersome process of measuring sow backfat thickness and promotes the automation of sow farms.

## Simple Summary

The backfat thickness of sows reflects their nutrient reserve levels. Controlling the backfat thickness within an appropriate range at different production stages can improve the production performance of sows. However, in large-scale pig farms, due to the cumbersome process of measuring backfat thickness and the large number of sows, it is often not possible to obtain or visually measure the backfat thickness during production. Often, a small difference in feed intake will lead to a large difference in production performance, such that sow production performance cannot be fully optimized. In this study, the relationships between backfat thickness and parameters related to hip area, hip width, hip height, and hip radius are discussed, and a sow backfat thickness estimation model is established. Finally, a sow backfat thickness estimation system is built using the LabVIEW 2023 Q1 (64-bit) software development platform. After inputting the sow buttock image, the system can automatically preprocess the image and extract the parameters required to quickly estimate the backfat thickness of the sow, thereby enhancing the automation of the sow farm.

## 1. Introduction

In large-scale farms, sows are the core of productivity. The reproductive performance of sows directly affects the economic efficiency of pig farms [1]. Research indicates that, during the breeding process, too much fat on sows can affect embryo implantation and lead to difficult labor. On the other hand, too little fat can result in weak uterine contractions during delivery and uneven litter sizes [2,3]. Therefore, maintaining sows within a reasonable body condition range helps to increase the number of pigs weaned per sow per year (PSY) and ultimately enhances the economic benefits of the pig farm [4,5]. Backfat thickness (BFT) reflects the fat depth and nutritional reserves of the sow [6]. In the production of sows, it is necessary to pay attention to changes in BFT over time to monitor their nutritional reserve status so as to make accurate adjustments to the feed ratio and thus improve their production performance and service life [7]. The BFT of sows is typically measured using a veterinary ultrasound device [8]. To improve the accuracy of the measurement, the skin at a point 6.5 cm from the midline of the back along the external cutting line of the last rib (P2 point) needs to be cleaned, and any hair should be removed. Then, coupling gel is applied to the probe of the ultrasound device to exclude any air between the probe and the pig’s body [9]. The measurement is obtained three consecutive times, and the average of these measurements is used as the final result. Each measurement should be taken while the pig is calm to avoid errors caused by the pig arching its back or sagging its waist. This method is not only time-consuming and labor-intensive, but it is also influenced by the experience of the operator and can easily cause stress to the pigs [10]. In traditional production, due to the large number of pigs and limited labor in large-scale pig farms, backfat measurements are often not performed during the rearing of sows, leading to a rough management approach. Even small differences in feed allocation can result in significant variations in production, which leads to the underutilization of the sow’s production potential.

In current research, methods based on machine vision technology for monitoring the body conditions of sows typically categorize their body condition into five grades: too lean, slightly lean, ideal, slightly fat, and too fat. If combined with BFT, the evaluation would be more effective [11,12,13,14]. However, there are very few studies that estimate sow BFT using machine vision technology [15] because measurement methods based on machine vision technology are easily affected by environmental changes, such as indoor lighting, pig movement, dust, and pen obstruction [16]. As a result, most current research still focuses on measuring morphological parameters such as body length, body width, body height, buttock width, and buttock height [17,18,19,20,21]. Moreover, the process of acquiring these parameters requires simultaneously capturing images of both the back and the side of the pigs. Placing vision systems on the sides of the pigs in a production setting can easily lead to obstruction by feces or even damage by the pigs, making it difficult to install and apply such systems in large-scale pig farms.

Research concerning the relationship between the BFT of sows and external morphological parameters has focused on associations with the eye muscle area (the cross-sectional area of the muscle between the first and second last thoracic vertebrae) and the depth of the eye muscle. A highly significant positive correlation between BFT and both the eye muscle area and the depth of the eye muscle has been reported [22]. Bin et al. conducted studies on the relationship between BFT and pig body weight, birth weight, and body condition score. The findings revealed a low correlation between body weight and BFT. In addition, the body condition score did not accurately reflect the reproductive performance of sows [23]. Piglets with a higher birth weight tend to have greater BFT [24]. The above research revealed correlations between the BFT of sows and the eye muscle area, eye muscle depth, body weight of the pigs, birth weight, and body condition score. However, measuring the eye muscle area and eye muscle depth is also time-consuming and labor-intensive, and it is difficult to automate these processes. Thus, these parameters are unsuitable for use in the development of a BFT estimation model for large-scale farms. The correlations between BFT and pig body weight, birth weight, and body condition score are not strong, leading to certain limitations when using these parameters to develop a BFT estimation model. Developing a BFT estimation model based on multiple external morphological parameters of sows is an effective approach to addressing this issue.

In organisms, the same genes may simultaneously control the expression of multiple traits, and these traits are often correlated. Therefore, some scholars have also investigated the correlations between BFT and other traits from the perspective of gene expression [25,26]. They found that in Landrace and Duroc pigs, BFT, daily weight gain, and hind leg weight are influenced by the same genes [27]. PCK1, ME1, ACLY, ACACA, FASN, and SCD were identified as key genes associated with back fat deposition [28]. In Landrace pigs, BFT, eye muscle area, intramuscular fat content, and lean meat percentage are controlled by the same gene [29]. Han et al. found that the MC4R gene has a significant impact on pig body weight, daily weight gain, eye muscle area, lean meat percentage, and BFT [30]. Wang et al. conducted principal component analysis, correlation analysis, and cluster analysis on eight traits in Yorkshire pigs: body weight, BFT, body height, body length, chest depth, chest width, girth, and hip circumference. The results indicated highly significant positive correlations among all these traits [31]. The above research demonstrates that a correlation exists between the BFT of sows and daily weight gain, hind leg weight, eye muscle area, intramuscular fat content, lean meat percentage, body weight, body height, body length, chest depth, chest width, girth, and hip circumference. However, few studies have established estimation models for sow BFT. There is limited research on the estimation of backfat thickness in sows. Only Teng et al. [32] developed a linear prediction model for sow BFT based on the curvature of the sows’ buttocks, with a maximum relative error of 7.71%. This method only examined the relationship between the curvature of the buttocks and BFT. If more external morphological parameters of sows could be incorporated, the accuracy of the predictions could potentially be further improved. Therefore, non-contact, automatic, real-time acquisition of sow BFT is crucial [19]. There is an urgent need to develop accurate and intelligent non-contact methods for continuous, real-time monitoring of sow BFT. This represents a crucial path for the healthy and sustainable development of pig farming in the future.

Due to their good maternal qualities, high litter size, strong adaptability, and high lean meat percentage, Landrace–Yorkshire crossbred sows have become the main force in piglet breeding in large-scale farms. This study aims to use machine vision technology, with Landrace–Yorkshire crossbred sows as the research subject, to investigate the correlations between BFT and multiple external morphological parameters such as buttock area and contour radius. This study also seeks to establish an estimation model for sow BFT and an automatic estimation system for its measurement, promoting the automation of sow farm management.

## 2. Materials and Methods

This study was conducted at Tian Sheng Yuan Farm from 5 January 2024 to 5 February 2024. The farm houses 154 multiparous Landrace–Yorkshire crossbred sows and is equipped with modern facilities, including automatic temperature control, automatic water dispensing, automatic feeding, and trench drainage systems. Feeding occurs twice daily at 8:00 and 15:00. Using large-batch sow production technology, the PSY is approximately 23. The farm uses natural gas for heating during the winter. During the experimental period, the average temperature in the pig houses was 23.6 °C (range: 18.2–25.7 °C), and the relative humidity ranged from 32% to 60% (average: 45%).

### 2.1. Data Acquisition

**Figure 1:**
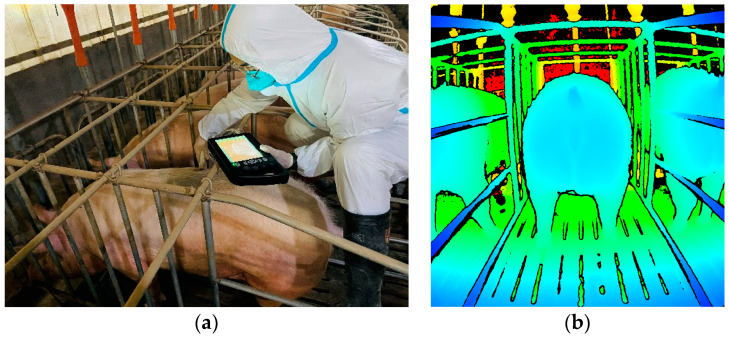
(**a**) Measurement of backfat thickness; (**b**) acquired depth image.

Since sows spend a considerable amount of time lying down each day, data acquisition was performed during each feeding. At this time, the indoor lighting conditions are also optimally sufficient, which enhances the quality of depth image acquisition. The BFT of each pig was measured using the Pig Doctor veterinary ultrasound backfat meter (Figure 1a) and its accompanying 3.5 MHz convex array probe, which has greater than 95% detection accuracy. Before the measurement, the hair at the P2 point was first removed, and the skin was cleaned to improve measurement accuracy. Then, coupling gel was applied to the BFT meter probe to eliminate air between the probe and the pig’s body. The measurement was taken three times consecutively, and the average value was used as the final result. BFT data were collected for all 154 sows on the farm. The number of sows with BFT in the ranges of 13–16 mm, 17–20 mm, 21–24 mm, and 25–29 mm were 24, 43, 48, and 39, respectively.

Simultaneously, the Azure Kinect DK camera was used to capture depth images of each sow’s buttocks (Figure 1b). After installing the Azure Kinect DK SDK on a laptop, the recorder supplied with the SDK can be used to capture depth images (https://learn.microsoft.com/zh-cn/previous-versions/azure/kinect-dk/azure-kinect-recorder, accessed on 26 June 2019). The standard deviation of random errors in the captured depth images is ≤17 mm, and the typical systematic error is <11 mm + 0.1% of the distance. Compared to Kinect V1 and V2, the Azure Kinect DK offers a significant improvement in resolution, resulting in depth images with less jagged edges and finer details, making it more suitable for describing the shape of a sow’s buttocks. During image acquisition, the camera was positioned parallel to the pig pen behind the pigs at a height of 95 cm above the ground. By setting the equipment at this height, we can prevent the rear fence from obstructing the imaging process. The captured depth images were 1024 × 1024 pixels in size and 16-bit grayscale. In the depth images, different colors represent the varying distances from different parts of the pig’s body to the camera, effectively reconstructing the contour of the pig’s buttocks.

### 2.2. Deep Image Processing and Extraction of Buttock Shape Parameters

**Figure 2:**
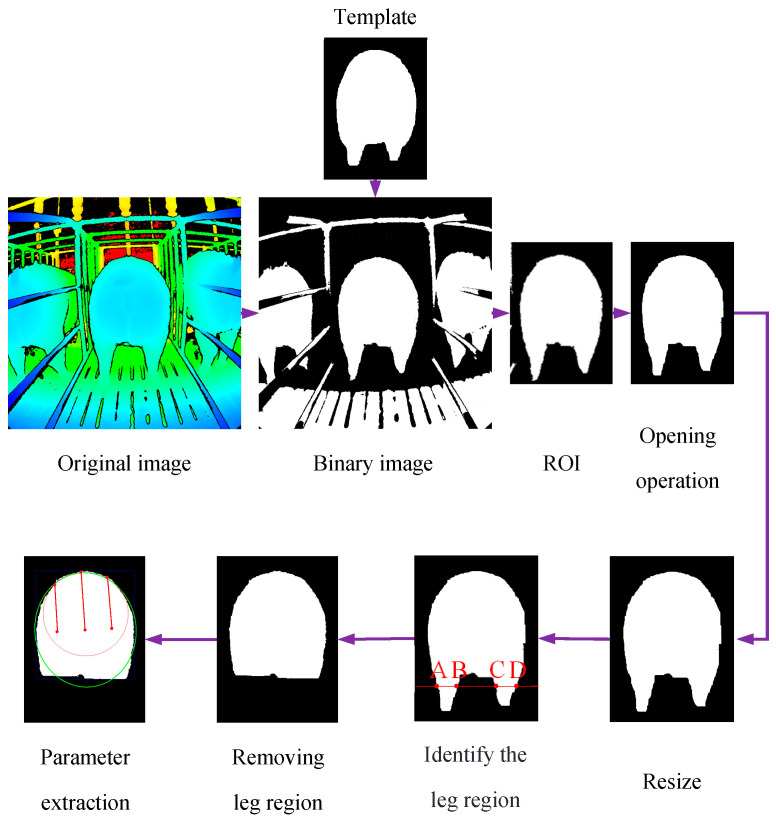
The depth image processing workflow.

Preprocessing the depth images of sows’ buttocks can reduce the impact of factors such as pen structures and jagged edges of pig bodies on the estimation results. Image preprocessing and parameter extraction were conducted using Python software (version 3.9.16) and the built-in image processing packages within the OpenCV library contained therein. The software offers a range of functionalities, including elliptical fitting, pixel area calculation, bounding rectangle fitting, line drawing, intersection determination, and circle creation. These features provide the necessary tools for the extraction of external morphological parameters. The depth image processing workflow of this study is shown in Figure 2. Due to the varying lengths of pigs’ tails and their instinctive swaying while feeding, which can affect the backfat estimation results, and to reduce the impact of the railings on the extraction of the sow’s area, binary processing was applied to the depth images of the sows’ buttocks. During binarization, since all the pig pens are the same size, images within the range of 400–800 mm are retained, while pixel values outside this distance range are set to 0. This process preserves the information within the region of the pig’s buttocks. To accurately extract the sow’s buttock area and minimize the influence of the stalls and neighboring sows on the estimation, template matching was used to refine the region extraction. During template matching, the original image of the pig’s buttocks is traversed from left to right and from top to bottom. The pixel-matching degree between the template and the overlapping sub-images is calculated from top to bottom. If the matching degree is higher, it indicates a greater likelihood of a match. The similarity calculation formula is provided below:

(1) $$R(x,y)=\frac{\sum_{x^{\prime },y^{\prime }}{\left(T(x^{\prime },y^{\prime })-I(x+x^{\prime },y+y^{\prime })\right)}^{2}}{\sqrt{\sum_{x^{\prime },y^{\prime }}T{\left(x^{\prime },y^{\prime }\right)}^{2}\cdot \sum_{x^{\prime },y^{\prime }}I{\left(x+x^{\prime },y+y^{\prime }\right)}^{2}}}$$

In the formula, *R*(*x*,*y*) represents the similarity at pixel (*x*,*y*) in the original image. A result closer to 0 indicates a higher match between the template and the image. *T*(*x*’,*y*′) represents the pixel value at position (*x*’,*y*’) in the template image. *I*(*x* + *x*′,*y* + *y*′) represents the pixel value at position (*x* + *x*′,*y* + *y*′) in the original image. After this step, only the sow’s buttock area and a small part of the stall area remained in the image. To further eliminate the stall area and smooth the image while minimizing jagged edges, a morphological opening operation was applied. After processing, the sow’s buttock area was completely extracted. Due to the variations in sow body length, the distance from the pig’s buttocks to the camera during image acquisition ranges from 500 mm to 700 mm. This variation in distance causes distortion in the size of the imaged contours, leading to inconsistencies in the captured images. To ensure that the sow’s buttock area is proportional to the size of the contour in the image, the image was scaled to 500 mm based on the distance from the pig’s buttocks to the camera. For example, if the distance from the sow’s hindquarters to the camera is 650 mm, the scaling ratio for the image is 1.3 (650 ÷ 500). Additionally, during feeding, sows often stand with one leg forward and the other backward, which can also affect the true contour size of the buttocks. To eliminate this effect, the leg regions in the image were removed by sequentially drawing horizontal lines from the bottom of the image upward and calculating the lengths of the line segments (AB, CD in Figure 2) formed between the horizontal lines and the sow’s body region. If the length was less than 130 pixels, it was determined to be a leg region. Subsequently, the edge pixels of the sow’s buttocks were extracted and fitted with an ellipse. First, the principal component analysis (PCA) algorithm was used to calculate the major and minor axis vectors of the point set and determine the angle of the major axis vector. Then, to minimize the sum of the squared distances from the points to the major axis vector, the lengths of the major and minor axes of the ellipse were calculated. Using the major axis vector and the lengths of the major and minor axes, a fitted ellipse was constructed. Finally, the ellipse was positioned and oriented through translation and rotation. In addition, the minimum bounding rectangle of the buttock contour was created.

In this study, a total of 10 parameters were extracted, including the area of the fitted ellipse (AFE), pixel area of the sow’s buttocks (AB), enclosing rectangle pixel width (RW), fitted ellipse major axis pixel length (EMA), fitted ellipse minor axis pixel length (EMI), fitted ellipse axis ratio (EAR), curvature of the sow’s buttocks (CB), enclosing rectangle pixel height (RH), aspect ratio of enclosing rectangle (ARR), and enclosing rectangle pixel area (RA). The method for extracting the sow’s buttock radius was as follows. Using the major axis of the fitted ellipse as the center, two parallel lines were drawn offset by 100 pixels to the left and right. A circle was then fitted through the three intersection points of these lines with the sow’s buttocks, and the radius of the circle was taken as the buttock radius. The correlation between the BFT and 10 external morphological parameters of 100 randomly selected sows was analyzed using SPSS data analysis software (version R26.0.0.0). Correlation and multiple linear regression analyses were conducted, and a sow BFT estimation model was established. The generalization performance of the model was evaluated using the remaining 54 sows that did not participate in the model construction and based on five aspects: determination coefficient (R^2^), mean absolute error (MAE), mean squared error (MSE), mean absolute percentage error (MAPE), and weighted mean absolute percentage error (WMAPE). MAE imposes a linear penalty for each prediction error, making it simple and intuitive, which is suitable for intuitive error analysis. In contrast, MSE assigns a larger penalty on bigger errors, making it more sensitive to large discrepancies. The calculation methods used for these metrics are shown in Equations (2)–(6).

(2) $$R^{2}=1-\frac{\sum_{i=1}^{n}{\left(y_{i}-{\hat{y}}_{i}\right)}^{2}}{\sum_{i=1}^{n}{\left(y_{i}-\bar{y}\right)}^{2}}$$

(3) $$MAE=\frac{1}{n}\sum_{i=1}^{n}\left|{\hat{y}}_{i}-y_{i}\right|$$

(4) $$MSE=\frac{1}{n}\sum_{i=1}^{n}{\left({\hat{y}}_{i}-y_{i}\right)}^{2}$$

(5) $$MAPE=\frac{1}{n}\sum_{i=1}^{n}\frac{\left|{\hat{y}}_{i}-y_{i}\right|}{y_{i}}$$

(6) $$WMAPE=\frac{\sum_{i=1}^{n}\left|{\hat{y}}_{i}-y_{i}\right|}{\sum_{i=1}^{n}y_{i}}$$

where *y_i_* is the measured BFT of the *i*-th sow, $\hat{y}$ *_i_* is the estimated BFT value of the *i*-th sow, and *n* is the total number of sows used for model validation.

## 3. Results

**Figure 3:**
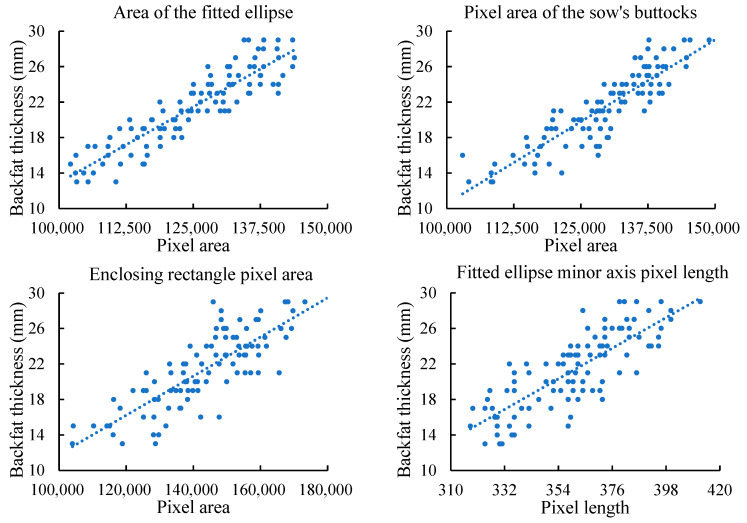

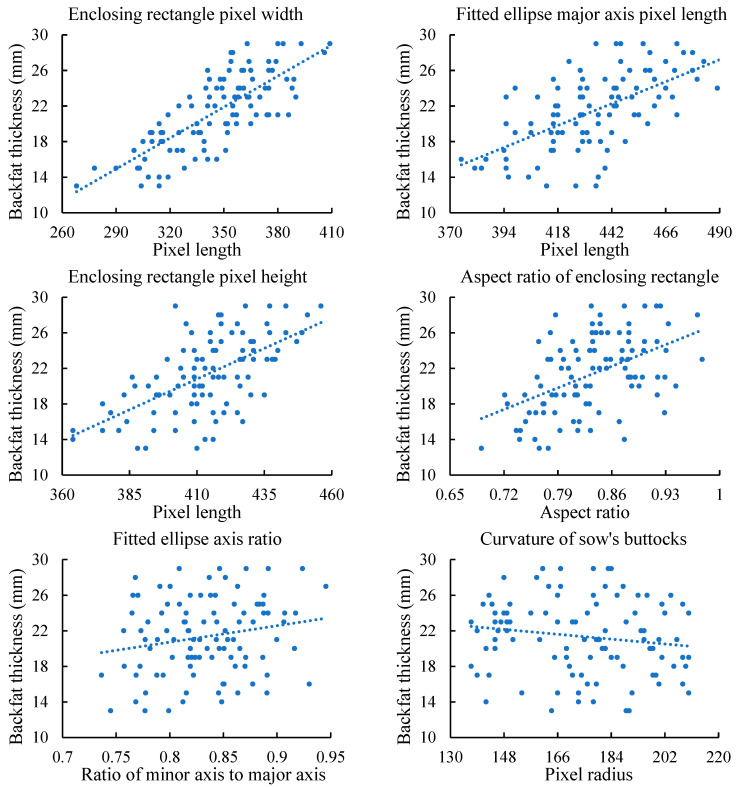
Relationship between sow backfat thickness and various external morphological parameters.

**Figure 4:**
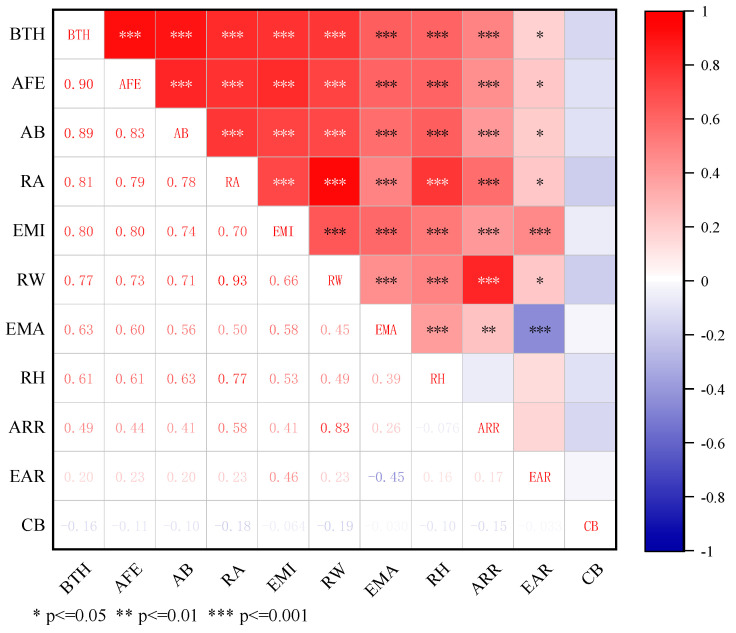
Heatmap of Pearson correlation coefficients between sow backfat thickness and various external morphological parameters.

The relationship between sow BFT and various external morphological parameters, along with the Pearson correlation coefficients heatmap, are shown in Figure 3 and Figure 4. The highest correlation is observed between sow BFT and parameters reflecting the size of the buttock area, followed by those reflecting the width of the buttock. The correlation with other parameters is relatively low. The Pearson correlation coefficients between BFT and the fitted ellipse pixel area, buttock pixel area, minimum bounding rectangle pixel area, the minor axis pixel length of the fitted ellipse, the width of the bounding rectangle, the major axis pixel length of the fitted ellipse, the height of the bounding rectangle, the aspect ratio of the rectangle, the ratio of the minor to the major axis of the fitted ellipse, and the buttock radius are 0.90, 0.89, 0.81, 0.80, 0.77, 0.63, 0.61, 0.49, 0.20, and −0.16, respectively.

A multiple linear regression analysis was conducted on the sow BFT and various external morphological parameters using SPSS data analysis software (version R26.0.0.0), and a sow BFT estimation model was constructed. A sow backfat thickness estimation model was constructed based on the area of the fitted ellipse (AFE), the pixel area of the sow’s buttocks (AB), the enclosing rectangle pixel width (RW), and the fitted ellipse major axis pixel length (EMA), as shown in Equation (7):

BFT = 0.00016 × AFE + 0.000161 × AB + 0.021884 × RW + 0.014466 × EMA − 33.22488(7)

The adjusted determination coefficient (R^2^) of the model was 0.889, indicating a good fit. The F-value was 198.63, with a *p*-value < 0.01, indicating that the model is statistically significant. The variance inflation factor (VIF) values for all estimated parameters were less than 5.0, indicating no multicollinearity among the selected parameters. Additionally, the Durbin–Watson statistic was 1.629, which falls within an acceptable range, further confirming the validity of the model.

**Figure 5:**
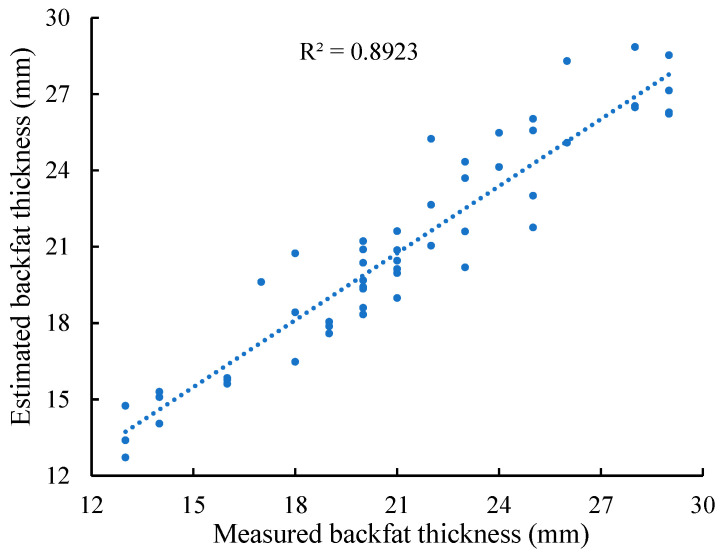
Comparison of estimated and measured backfat thickness for 54 sows not involved in model construction.

To evaluate the model’s generalization performance, it was tested using data from an additional 54 sows that were not involved in model construction. The estimated BFT versus the actual BFT is shown in Figure 5. A determination coefficient (R^2^) of 0.8923, a mean absolute error (MAE) of 1.23 mm, a mean squared error (MSE) of 2.26 mm^2^, a mean absolute percentage error (MAPE) of 5.73%, and a weighted mean absolute percentage error (WMAPE) of 5.78% were obtained. These metrics suggest that the model performs well and can provide accurate and reliable estimates of sow BFT.

**Figure 6:**
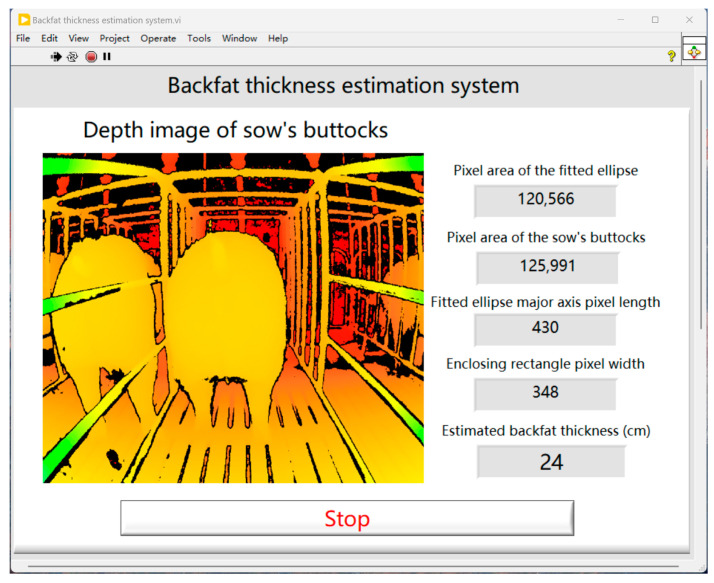
Sow backfat thickness estimation system.

Based on NI LabVIEW 2023 Q1 (64-bit) software, a sow BFT estimation system was developed, as shown in Figure 6. The built-in Python nodes and image-processing modules in the software were useful for system development. After inputting images of the sows’ buttocks, the system can automatically perform a series of preprocessing steps, including binarization, template matching, opening operations, scaling, removal of leg regions, and ellipse fitting. These preprocessing steps are designed to extract the required parameters for BFT estimation, such as the pixel area of the fitted ellipse, buttock pixel area, major axis length of the fitted ellipse, and width of the bounding rectangle. The system then completes the automatic and real-time estimation of sow BFT without any human intervention.

## 4. Discussion

In sow breeding, the stockpersons usually estimate the body condition and BFT of sows visually. They believe that sows with large and broad hips have greater BFT and better nutritional reserves. The results of this study show that the Pearson correlation coefficients between sow BFT and parameters related to buttocks area are higher than 0.8, and those related to buttock width are higher than 0.77, which is consistent with practical experience. This conclusion is also consistent with the research findings of Wang et al. [31]. The ellipse fitting method can provide a more accurate assessment of a sow’s BFT. This might be due to the fact that the sow was feeding when the image of the buttocks was captured. While feeding, the sow’s body relaxes and sways, which can cause distortion in the imaging of the pig’s buttocks. Additionally, the lighting conditions within the pen may also affect the final image, leading to variations in the pixel area size of the buttocks, thus producing an error. Ultimately, this causes the correlation between the fitted ellipse area and the short axis length to be superior to that of other similar parameters. The parameters reflecting the height of the sow, such as the major axis length of the fitted ellipse and the height of the bounding rectangle, show lower correlations. This may be because, although increased BFT can lead to an increase in body height, the inherent height differences among individual sows limit the strength of this correlation. Even sows of the same breed exhibit variable height proportions. Taller sows might not necessarily have more backfat compared to shorter sows, as backfat accumulation is influenced by multiple factors, including genetics, nutrition, and stage of gestation or lactation. Therefore, this also results in a lower correlation between the aspect ratio of the rectangle and BFT.

The results of this study indicate that the correlation between BFT and parameters other than the buttock radius is significant. This conclusion is also consistent with the reference [31]. In the study by Teng et al., the curvature of the sow’s back exhibited a strong correlation with BFT [32]. Some sows exhibited a twisted body posture during image acquisition in this study, causing image distortion. This led to errors in the extracted buttock radius, resulting in a lower correlation between the buttock radius and BFT. In practice, obtaining a standard image of the sow’s buttocks is challenging; thus, the method of estimating BFT from buttock images with the legs removed is more practical.

The determination coefficient (R^2^) between the estimated and measured BFT using the model developed in this study is 0.8923, indicating greater accuracy compared to methods relying exclusively on pixel area. This suggests that using the pixel area of the fitted ellipse, the pixel area of the sow’s buttock, the width of the bounding rectangle, and the major axis length of the fitted ellipse can complement each other and overcome the errors associated with single-factor estimation. The Durbin–Watson statistic for the sow BFT estimation model developed in this study is 1.629. This value is somewhat below the ideal value of 2.0, possibly due to the fact that all pigs in the study were from the same breed and had similar feeding times, feed, and growth environments. Nevertheless, this value is still within an acceptable range.

The estimation errors may originate from the following factors:

(1) Variations in sow posture can lead to changes in the actual hip area, fitted ellipse, and bounding rectangle, ultimately resulting in estimation errors.
(2) The inherent inaccuracies of the depth camera could result in estimation errors. In this study, the imaging distance was controlled within a range that minimizes the camera’s error to reduce such inaccuracies.
(3) Dim lighting conditions within the pen can result in a decrease in image quality, leading to an increased error in defining the sow’s contour edges, thus causing estimation errors.
(4) Individual differences among sows may cause estimation errors. Some sows have wider bone structures, which may appear as larger hip areas and body widths in images but actually have thinner BFT.

The sow backfat thickness estimation system developed based on NI LabVIEW 2023 Q1 (64-bit) software can achieve real-time automatic estimation of BFT. The deployment of this system is designed to reduce the requirement for at least one, and potentially more, staff members dedicated to assessing the body condition of sows. It also eliminates the subjective inaccuracies often associated with visual assessments by personnel. By providing management with timely insights into the physical status of each individual sow, the system helps prevent feed inefficiencies. With precise evaluations of backfat thickness, timely adjustments to nutritional intake can be made, improving the reproductive performance of sows and enhancing overall operational efficiency and economic benefits for the farm.

## 5. Conclusions

This study investigated the relationship between sow BFT and external morphological features using stereovision technology. The BFT of sows shows the strongest correlation with parameters characterizing the buttock area and width, particularly with the pixel area of the fitted ellipse and the pixel length of its short axis. Correlations with other parameters are relatively weak. An estimation model was constructed using the morphological parameters of the sow’s buttocks to accurately estimate the BFT of sows. The sow backfat thickness estimation system, built on the LabVIEW 2023 Q1 (64-bit) software development platform, is designed to automatically perform preprocessing on captured images of the sow’s hindquarters and immediately extract the required parameters for estimating backfat thickness. This process facilitates the quick and precise measurement of sow backfat thickness, streamlining its assessment and enhancing efficiency.

The above results indicate that machine vision technology can be used to estimate backfat thickness in sows. This technology can be integrated into inspection robots used in pig farms to achieve real-time acquisition of sow backfat thickness. This integration would reduce labor costs while eliminating the time-consuming, labor-intensive processes and minimizing the significant subjective errors associated with manual measurements. Additionally, it would facilitate making real-time adjustments to sow nutrition, potentially increasing litter size and the number of healthy piglets and improving piglet growth rates. Ultimately, this would enhance the PSY metric for the farm, thereby boosting economic efficiency. Further research can be conducted to explore the relationships between the area-related parameters of sows and BFT, such as the area of the back, aiming to develop more accurate and efficient sow BFT estimation techniques. This study only examined the backfat thickness of sows from a single breed raised in the same environment. To generalize this model to other breeds of sows, it may be necessary to collect sufficient data from the target breeds to fine-tune the estimation results.

## Data Availability

If interested in the data used in the research work, contact zhjl@sxau.edu.cn for the original dataset.
